# Around the Hospitals

**Published:** 1895-07-27

**Authors:** 


### Around the Hospitals.

[Notice to the Managers of Institutions.—Special space is now reserved for the insertion of notices of hospital meetings and festivals. The Editor requests that all notices may reach The Hospital Office, 428, Strand, at least one week before the date of each meeting.]

**The Sydney Hospital.**—The number of patients under treatment was 3,508. Urgent cases admitted, 1,401. Under Government orders 1,197 were admitted, and 486 contributed more or less towards their treatment. The remainder were admitted under subscribers' orders. The average duration of stay was 26·11 days. The death-rate was 11·8. Of the 297 deaths occurring, 111 died within twenty-four hours of admission. The attendance of patients at the various outdoor departments, including two outlying district departments, was 113,968.

**The Hampstead Home Hospital.**—Since the issue of the last report of this institution changes have been made in its constitution, and it is no longer only a pay hospital, since free patients are received. According to the new arrangements the hospital has now three private wards, one isolation ward, two men's, three women's, one children's ward, and an accident ward. Naturally the admission of free patients has affected the finances, and patients' payments fell in 1894 from £628 in the previous year to £410. That the alteration is regarded as fulfilling a need is, however, shown by the increased amount of annual subscriptions and donations, which bring the income of 1894 up to £3,288, against £2,076 received in 1893. Free patients have been admitted since May, 1894; to December of the same year 107 had been received, nearly half the total number of 221 in-patients. The out-patients numbered 177. The income for the past year amounted to £3,288, and the expenditure to £3,004. It must not be overlooked that the festival of 1894 is accountable for the adequate income of this year, and that much larger subscriptions will be necessary to finance the current year.

